# Endothelial Redox Dysregulation in Pulmonary Arterial Hypertension: Compartmentalized Mechanisms and Therapeutic Implications

**DOI:** 10.3390/ijms27209096

**Published:** 2026-10-14

**Authors:** Santiago Moreno-Caceres, David P. Marciano

**Affiliations:** 1 Center for Translational Science, Florida International University, Port Saint Lucie, FL 34987, USA; 2 Department of Cellular & Molecular Medicine, Herbert Wertheim College of Medicine, Florida International University, Miami, FL 33199, USA

**Keywords:** pulmonary arterial hypertension, endothelial dysfunction, oxidative stress, reactive oxygen species, redox signaling, NADPH oxidase, mitochondria, nitric oxide, mechanotransduction, circulating biomarkers

## Abstract

Pulmonary arterial hypertension (PAH) is a progressive pulmonary vasculopathy in which endothelial dysfunction drives increased pulmonary vascular resistance and arterial remodeling. Redox signaling is central to endothelial homeostasis, and its disruption in PAH reflects changes in the magnitude, source, and subcellular location of reactive oxygen species (ROS) production, as well as the molecular targets they engage. In healthy endothelium, compartmentalized redox signaling supports adaptive responses while preserving nitric oxide bioavailability. Loss of this spatial control in PAH can convert normally transient redox signals into persistent endothelial dysfunction, although the strength of evidence for this conversion differs substantially between pathways. This review examines how disease-associated stressors reshape endothelial redox signaling through NADPH oxidases, mitochondria, eNOS, iron-dependent chemistry, and nuclear pathways and how these compartments communicate. We also examine emerging circulating markers of redox and endothelial dysfunction and evaluate redox-directed therapies across experimental pulmonary hypertension and PAH. Throughout, we grade each mechanism according to whether it is supported by evidence from human PAH vascular tissue, patient-derived pulmonary arterial endothelial cells, experimental pulmonary hypertension models, and pulmonary endothelial models or by extrapolation from other relevant disease systems, and we identify where compartment-specific evidence is inferred from enzyme localization rather than measured directly. Current evidence supports moving beyond broad antioxidant approaches toward strategies that selectively target pathogenic redox signaling within the relevant endothelial compartment.

## 1. Introduction

Endothelial dysfunction is a central driver of pulmonary arterial hypertension (PAH), contributing to disordered angiogenesis and altered inflammatory, thrombotic, and paracrine signaling within the vascular wall [1,2,3,4,5,6]. These changes promote remodeling of small pulmonary arteries, increased pulmonary vascular resistance, and ultimately right ventricular failure. Rather than simply reflecting elevated pulmonary pressure, endothelial dysfunction contributes directly to PAH onset and progression.

Oxidative stress is a central component of endothelial dysfunction in PAH and reflects disruption of redox homeostasis across distinct cellular sources and compartments rather than a uniform increase in reactive oxygen species (ROS). ROS and reactive nitrogen species are normal signaling intermediates whose effects reflect where they arise, how long they persist, and which molecular targets they engage. A transient H_2_O_2_ signal near the plasma membrane is biologically distinct from sustained mitochondrial superoxide, peroxynitrite generated by uncoupled eNOS, or hydroxyl radical formed in the presence of redox-active iron [7,8]. Kinetic considerations reinforce this distinction because the targets an oxidant actually reaches are set by local reaction rates and restricted diffusion rather than by bulk concentration [9]. Oxidative stress is also evident in lungs from patients with severe pulmonary hypertension [10], providing direct human evidence of oxidative injury without identifying its cellular source or subcellular compartment.

Evidence for these redox mechanisms spans human PAH tissue, patient-derived endothelial cells, and experimental pulmonary hypertension and broader endothelial models, and these levels of support are not interchangeable. Throughout this review, we distinguish findings in human PAH vascular tissue, findings in pulmonary arterial endothelial cells (PAECs) isolated from patients with PAH, findings in experimental pulmonary hypertension, findings in pulmonary endothelial models without a PAH genotype or exposure, and mechanisms extrapolated from non-pulmonary or non-endothelial systems. Table 1 classifies the pathways discussed here on this basis, and the prose identifies the system in which each principal observation was made. This review organizes endothelial redox dysfunction by oxidant source and subcellular compartment rather than by global ROS abundance and treats the strength and disease specificity of the supporting evidence as a property of each individual pathway rather than of the framework as a whole. We also note where the compartmentalization framework rests on measured compartment-specific oxidant signals and where it rests on inference from redox enzyme localization.

## 2. Pulmonary Arterial Hypertension and Endothelial Dysfunction

Endothelial dysfunction in PAH extends beyond impaired vasodilation and reflects a broader loss of vascular homeostasis. Reduced nitric oxide bioavailability [24] occurs alongside abnormal endothelial proliferation, apoptosis resistance, disordered angiogenesis, barrier dysfunction, and altered inflammatory and thrombotic signaling [1,2,3,4,5,6]. These changes contribute to intimal remodeling, plexiform lesion formation, luminal narrowing, and eventual vascular obliteration [76]. Rather than a single endothelial defect, PAH involves coordinated disruption of multiple endothelial programs that normally regulate vascular tone, structural integrity, and communication within the pulmonary arterial wall.

Genetic evidence further supports a central endothelial role in PAH, with BMPR2 as the most common heritable genetic cause [5,6]. Loss of BMPR2 signaling increases susceptibility to oxidative injury [77], impairs mitochondrial recovery after stress [18], and alters endothelial substrate use and glutamine dependence [78,79]. Restoring BMPR2 signaling can reverse endothelial dysfunction and experimental pulmonary hypertension [80], while BMPR2 also restrains excessive TGF-β signaling and abnormal endothelial mechanics [81].

Pulmonary endothelial cells continuously integrate mechanical and metabolic cues from the circulation, including shear stress, pulsatility, and oxygen tension. Under physiologic conditions, laminar shear stress maintains endothelial quiescence, nitric oxide signaling, and vascular homeostasis [57]. Disturbance of either mechanical or oxygen-dependent signaling can contribute to pulmonary vascular dysfunction. These disturbances arise in different disease contexts, with chronic hypoxia promoting vascular remodeling [82]. In congenital heart disease-associated PAH (CHD-PAH), a left-to-right shunt exposes the pulmonary circulation to elevated blood flow, altered shear and pulsatile forces, and higher arterial oxygen content distal to the shunt [83]. Arteries from patients with congenital heart defects and pulmonary hypertension show prominent endothelial abnormalities [84], supporting the concept that altered hemodynamics and oxygen tension can converge on the endothelium to promote disease.

The endothelial abnormalities described in this section are among the best supported in PAH, resting on human lung tissue, patient-derived cells, and human genetics. The redox mechanisms proposed to underlie them, considered in the sections that follow, are supported far less evenly, and we state the level of evidence for each.

## 3. Physiologic Redox Signaling in the Pulmonary Endothelium

### 3.1. Oxidative Signaling Versus Oxidative Stress

Endothelial redox homeostasis depends on the magnitude, source, duration, and subcellular localization of oxidant production. Physiologic H_2_O_2_ and superoxide modify redox-sensitive proteins and couple environmental cues to cellular responses [8], while peroxiredoxins and related systems limit the distribution and duration of these signals [85]. Oxidative stress develops when this spatial and temporal organization is lost and oxidant production becomes sustained or extends beyond its normal compartment. Global ROS measurements do not capture this distinction because they cannot resolve whether oxidant production is transient and localized or sustained within specific compartments, nor can they identify the molecular targets these signals engage. In PAH, this context is critical because the biological effect of ROS depends on where they are generated, how long they persist, and which signaling pathways they modify.

### 3.2. Redox Microdomains and Compartmentalized Signaling

Redox compartmentalization refers to confining an oxidant signal to a defined subcellular volume on a timescale set by its production rate and the local capacity to remove it. Its loss is not a single process, and we use the term to cover four distinguishable changes. The first is redistribution of oxidant production, in which an oxidase is expressed, assembled, or trafficked to a particular compartment. The second is altered local scavenging capacity, in which peroxiredoxin, glutathione peroxidase, catalase, or thioredoxin activity falls within a compartment, allowing a signal that would normally be quenched to instead persist and diffuse to neighboring compartments. The third is altered transmembrane transport, principally aquaporin-facilitated movement of H_2_O_2_ between compartments. The fourth is source-to-source amplification, in which oxidants generated in one compartment stimulate production in another. These mechanisms are not mutually exclusive and are rarely separated experimentally, but they carry different therapeutic implications and should not be treated as a single phenomenon.

NADPH oxidases illustrate the first mechanism because they generate ROS at defined membrane and intracellular sites, including plasma membrane domains, endosomes, the endoplasmic reticulum (ER), and perinuclear regions [7,86]. This localization lets oxidants regulate nearby proteins without causing a large increase in global ROS. In PAECs from patients with PAH, endothelial NOX1 assembly drives a proliferative Gremlin-1 pathway [12], illustrating how the identity and localization of the active oxidase can shape downstream signaling.

Transport and buffering determine how far such a signal travels. Aquaporins regulate H_2_O_2_ diffusion between compartments, with AQP3 and related channels allowing locally generated peroxide to reach intracellular targets, although this has been established in non-pulmonary cells [64,65]. Mitochondria provide another major redox compartment and communicate bidirectionally with NADPH oxidases. NOX-derived ROS can stimulate mitochondrial ROS production, while mitochondrial ROS production can activate cytosolic and membrane oxidant pathways [66]. This crosstalk allows a localized redox signal to spread without requiring uniform oxidation of the cell, and Section 6 discusses it in detail.

The nucleus maintains a distinct redox environment in which glutathione, thioredoxin, peroxiredoxins, and APE1/Ref-1 regulate transcription, DNA repair, and chromatin structure and function [87]. Oxidative DNA modification can also support redox-responsive transcription, with OGG1 recognizing 8-oxoG in regulatory regions and influencing transcription through both repair-dependent and noncanonical signaling mechanisms [43]. OGG1 can function together with APE1/Ref-1 at oxidized promoters, linking base excision repair to transcriptional regulation [43]. NOX4 has also been localized to the nucleus and linked to redox-responsive transcription, although this was shown in human umbilical vein endothelial cells rather than in pulmonary endothelium [42]. Although that study reported nuclear superoxide, biochemical work identifies H_2_O_2_ as the dominant product released by NOX4 [88]. Whether nuclear NOX4 is present or active in pulmonary endothelial cells, and how oxidized DNA and associated repair proteins contribute to human PAH, remains unresolved.

A distinction should be drawn between compartment-specific oxidant signals that have been measured and those inferred from redox enzyme localization. Genetically encoded probes targeted to individual compartments can now resolve H_2_O_2_ at physiologic concentrations and have demonstrated genuine intracellular gradients, including diffusion of H_2_O_2_ from the mitochondrial matrix [89]. These approaches have not been applied to pulmonary endothelial cells from patients with PAH. Accordingly, most statements about compartment-specific redox signaling in PAH, including those in this review, are inferences drawn from where redox enzymes are located, which targets are modified and which reactive species a given source generates, rather than from direct measurement of compartmentalized oxidant signals in diseased human pulmonary endothelium. We identify this inference explicitly where it occurs. Compartment-targeted redox sensors and spatially resolved redox proteomics in pulmonary vascular endothelium should allow more direct testing of these relationships in PAH specifically.

### 3.3. Laminar Shear Stress and Endothelial Redox Homeostasis

Physiologic laminar shear stress promotes endothelial alignment and vasoprotective transcription through redox-sensitive pathways. KLF2 and KLF4, two Krüppel-like transcription factors, support endothelial quiescence [57,58] and are reduced in human PAH [53,54]. Endothelial KLF4 loss also worsens experimental pulmonary hypertension [55], linking disruption of this shear-responsive program to disease.

Redox signaling also helps couple shear stress to endothelial adaptation, as transient ROS can activate NRF2, while NOX4-derived H_2_O_2_ promotes shear-dependent sulfenylation of the phosphatase SHP2 and eNOS activation [15,90]. Localized oxidant production can support a physiologic shear response, while changes in its magnitude or location may shift the same pathways toward endothelial dysfunction.

The shear-responsive KLF2/KLF4 program also extends to chromatin regulation, where KLF4 recruits the SWI/SNF chromatin remodeling complex and increases enhancer accessibility under laminar shear stress [51]. p300 activation supports NOS3 transcription, the gene encoding eNOS [59], while disturbed shear can alter endothelial DNA methylation through pathways dependent on DNA methyltransferases [60,61]. Although these mechanisms have been defined largely in systemic endothelial models, they strongly support the concept that hemodynamic forces influence endothelial chromatin state and transcriptional control.

### 3.4. Antioxidant Defense Systems

Endothelial antioxidant systems shape redox signaling by controlling the magnitude, duration, and localization of oxidant signals rather than simply removing ROS. Superoxide dismutases convert superoxide to H_2_O_2_, while catalase, glutathione peroxidases, thioredoxin systems, and peroxiredoxins control peroxide within specific compartments [85]. Because peroxiredoxins are abundant, fast, and unevenly distributed, they set the effective diffusion distance of H_2_O_2_ rather than simply its total concentration [9,85]. A local fall in peroxiredoxin or glutathione peroxidase capacity therefore extends the diffusion distance of an otherwise unchanged global oxidant burden.

Nuclear factor erythroid 2-related factor 2 (NRF2) broadens the transcriptional response to oxidative stress by modifying cysteines in its inhibitor, Kelch-like ECH-associated protein 1 (KEAP1), and inducing transcription of antioxidant and cytoprotective genes [91]. The glutathione system provides a major soluble redox buffer, with NADPH-dependent regeneration of reduced glutathione supporting glutathione peroxidase activity and peroxide detoxification [92]. Local oxidative stress can persist despite NRF2 activation, so antioxidant gene expression alone does not restore redox homeostasis and should be interpreted alongside compartment-specific measurements.

Redox signaling in the healthy pulmonary endothelium is therefore defined by where and for how long an oxidant acts, rather than by how much is present. This is why global measures of oxidative stress have limited explanatory value in PAH and why the remainder of this review is organized by oxidant source and subcellular compartment (Figure 1).

**Figure 1:**
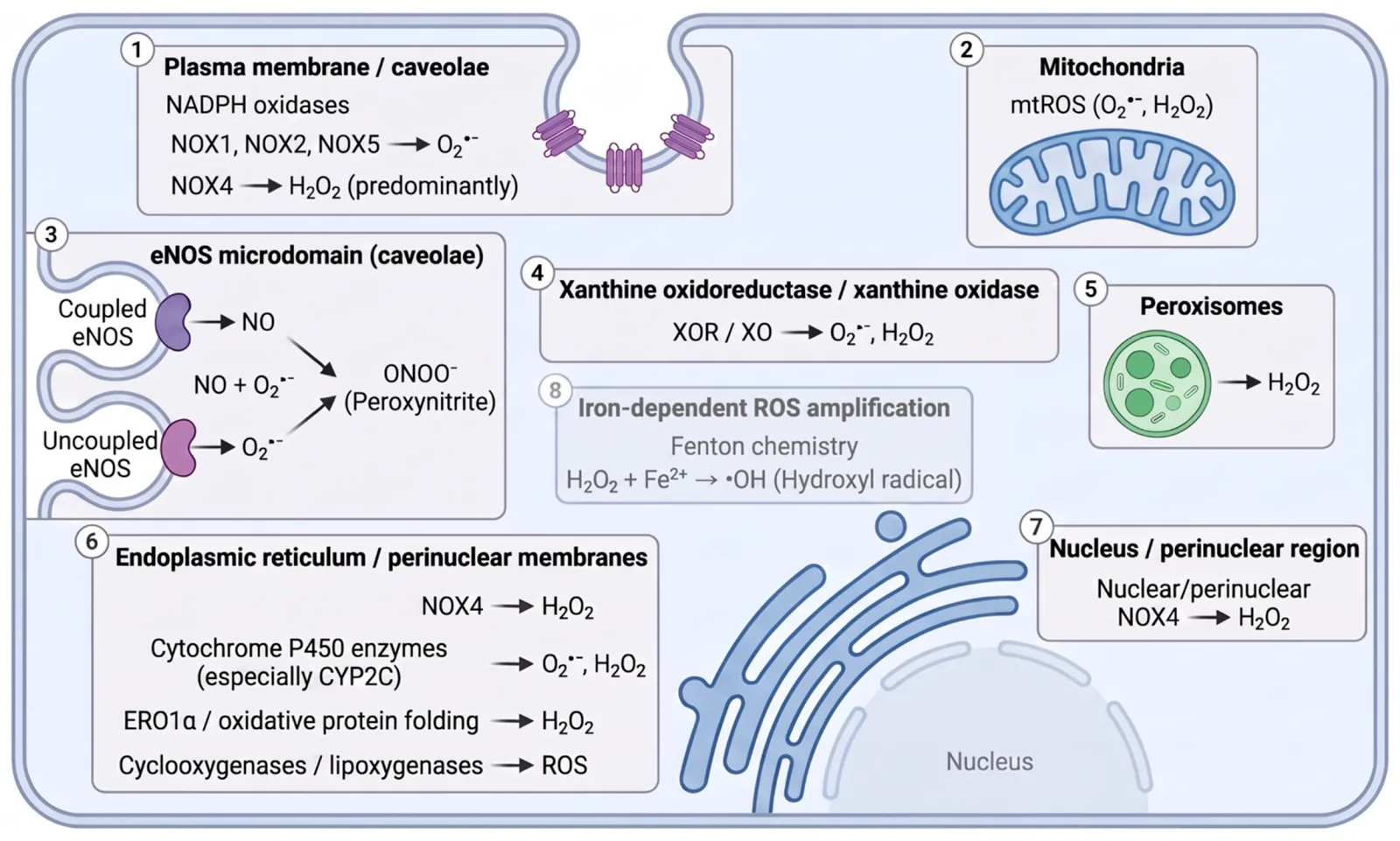
Compartmentalized sources of reactive oxygen species in pulmonary endothelial cells. Endothelial redox signaling arises from spatially distinct enzymatic and chemical sources that generate different reactive oxygen and nitrogen species. (1) At the plasma membrane and within caveolae, NADPH oxidases contribute to oxidant production: NOX1, NOX2, and NOX5 primarily generate superoxide (O_2_^•−^), whereas NOX4 predominantly produces H_2_O_2_. (2) Mitochondrial electron transport generates O_2_^•−^, which can be converted to H_2_O_2_. (3) Within caveolar eNOS microdomains, coupled eNOS produces nitric oxide (NO), whereas eNOS uncoupling favors O_2_^•−^ generation; reaction of NO with O^2•−^ produces peroxynitrite (ONOO^−^). (4) Xanthine oxidoreductase/xanthine oxidase contributes both O_2_^•−^ and H_2_O_2_. (5) Peroxisomal oxidases generate H_2_O_2_, which is normally constrained by antioxidant systems including catalase. (6) The endoplasmic reticulum and associated perinuclear membranes contribute oxidants through NOX4, cytochrome P450 enzymes, ERO1α-dependent oxidative protein folding, and cyclooxygenase/lipoxygenase pathways. (7) Nuclear and perinuclear NOX4 provide a potential local source of H_2_O_2_ capable of influencing redox-sensitive nuclear processes. (8) Labile ferrous iron can amplify oxidative chemistry through Fenton reactions, in which H_2_O_2_ is converted to a highly reactive hydroxyl radical (^•^OH). Together, these sources show that endothelial redox signaling depends on the identity, location, and local concentration of reactive species rather than a uniform intracellular ROS pool. Created in BioRender. Marciano, D. (2026) https://BioRender.com/0vfwdws.

## 4. Environmental, Hemodynamic, and Inflammatory Drivers of Endothelial Redox Dysregulation

### 4.1. Hypoxia, Increased Oxygen Exposure, and Dysregulated Oxygen Signaling

Hypoxia is a well-established driver of pulmonary vascular remodeling and illustrates how reduced oxygen tension can enhance specific redox pathways rather than simply suppress oxidative chemistry. During hypoxia, mitochondrial complex III-derived ROS contribute to stabilization of hypoxia-inducible factor (HIF) [21,22], and endothelial HIF-2α serves as a major oxygen-sensitive transcriptional mediator of pulmonary vascular remodeling [82]. Hypoxia also alters NADPH oxidase activity, nitric oxide signaling, and NOX4 expression in the pulmonary vasculature [93,94]. These responses demonstrate how changes in oxygen tension are integrated across mitochondrial, NOX-dependent, and nitric oxide pathways rather than through a single oxygen-sensitive mechanism.

Increased pulmonary arterial oxygen exposure can accompany abnormal hemodynamic load in CHD-PAH, although its independent contribution to endothelial redox dysfunction remains unresolved. In the ovine aortopulmonary shunt model, elevated pulmonary blood flow and pulmonary arterial oxygen tension are present before overt vascular remodeling develops [95,96]. Endothelial cells isolated from these animals show impaired eNOS signaling and evidence of uncoupling [97]. These findings identify increased oxygen exposure as one component of the shunt environment that may influence endothelial redox state but do not separate its effects from simultaneous changes in flow, shear, and pulsatility.

### 4.2. Abnormal Shear Stress and Hemodynamic Forces

The magnitude and pattern of shear stress strongly influence endothelial redox signaling. Physiologic laminar shear activates vasoprotective and antioxidant pathways, whereas oscillatory shear promotes p47phox-dependent NADPH oxidase activation, inflammatory signaling, and monocyte adhesion [62,63]. Much of this mechanistic evidence comes from systemic vascular models and should be interpreted as a demonstration that changes in mechanical force can alter endothelial redox state, rather than as direct evidence in the pulmonary vascular circulation.

The hemodynamic environment in PAH is spatially heterogeneous across the pulmonary arterial tree. Patient-specific computational modeling showed that wall shear stress decreases in the proximal pulmonary arteries with increasing disease severity but rises substantially in distal small arteries [98]. This distinction matters because the distal vessels most affected by remodeling may experience a markedly different mechanical environment than the proximal circulation. Excessive shear can directly alter endothelial identity, as high shear reduces expression of the transcription factor ERG and promotes endothelial-to-mesenchymal transition in a PAH-relevant aortocaval shunt mouse model [56], providing a mechanistic link between abnormal hemodynamic load and loss of endothelial phenotype.

### 4.3. Inflammatory Cytokines and Immune Activation

Inflammation and redox dysregulation are closely interconnected in PAH. Perivascular immune cell accumulation and elevated inflammatory cytokine signaling are prominent features of diseased pulmonary arteries in human tissue [99,100], while many inflammatory pathways both generate ROS and respond to changes in cellular redox state [6]. Activation of endothelial NF-κB and AP-1 increases adhesion molecule and cytokine expression [101], promoting leukocyte recruitment and reinforcing the interaction between inflammation and oxidant production.

Redox regulation is also embedded directly within endothelial inflammatory and barrier pathways. Recruitment of the adaptor protein Nck to PECAM-1 couples oxidative stress to canonical NF-κB activation [102]. In addition, the kinase TNIK promotes TNF-dependent phosphorylation of ezrin/radixin/moesin proteins and increases endothelial permeability, yet is reversibly inhibited by H_2_O_2_-mediated oxidation of a single cysteine [103]. These mechanisms show how specific redox-sensitive proteins translate changes in oxidant production into inflammatory and barrier responses, underscoring that the biological consequences of ROS depend on the molecular targets they engage rather than on total cellular ROS alone.

Hypoxia, altered oxygen exposure, abnormal shear, and inflammatory signaling therefore converge on the same endothelial oxidant sources, so the redox phenotype of a pulmonary artery in PAH reflects its local hemodynamic and inflammatory environment as much as any intrinsic cellular abnormality (Figure 2). This convergence is supported by human hemodynamic modeling and by experimental models, but not yet by direct measurement in diseased human pulmonary vessels.

**Figure 2:**
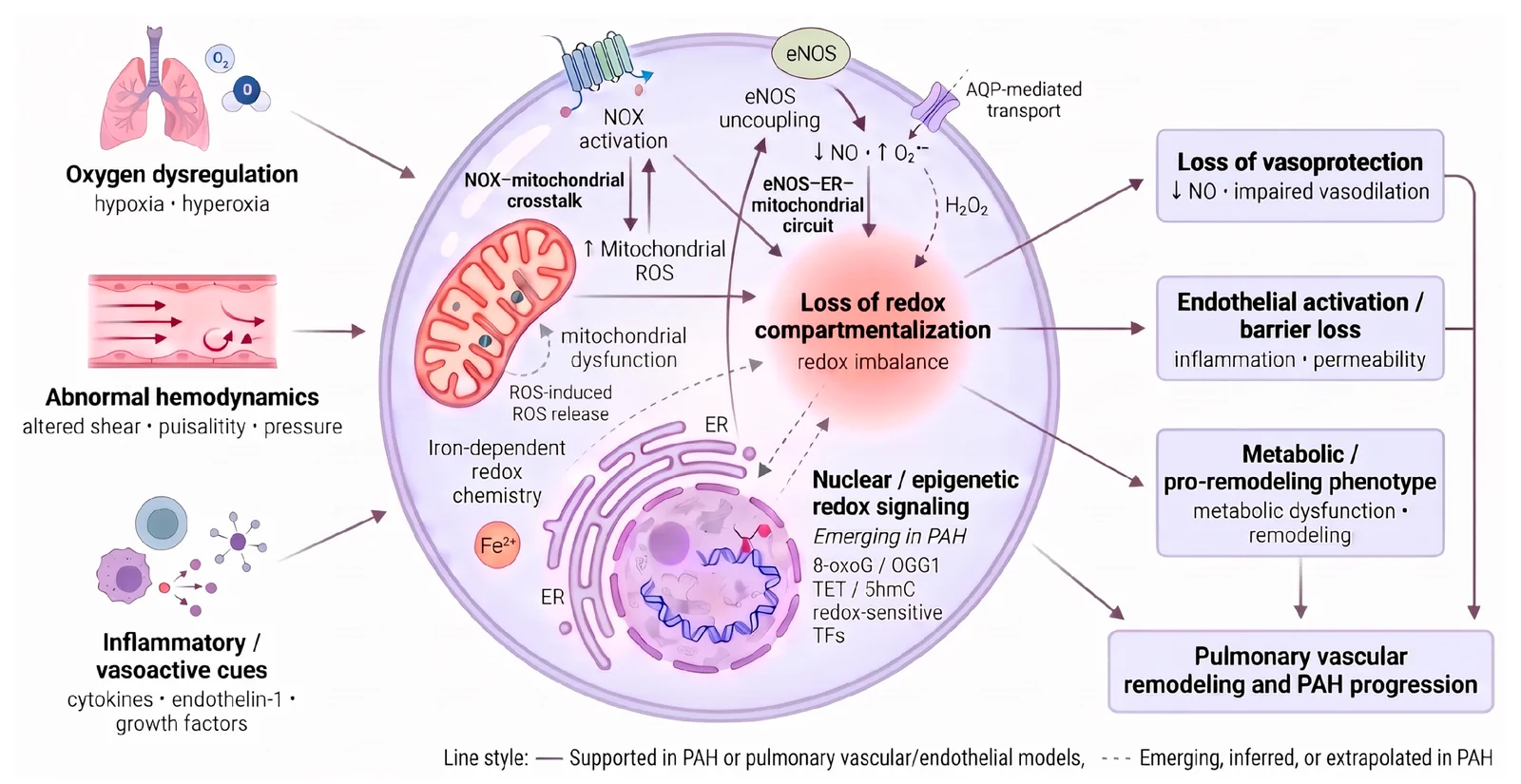
PAH-relevant stressors disrupt compartmentalized endothelial redox signaling and promote intercompartmental crosstalk. Oxygen dysregulation, abnormal hemodynamics, and inflammatory or vasoactive cues alter redox signaling across plasma membrane/caveolar, mitochondrial, endoplasmic reticulum, iron-dependent, and nuclear compartments. Crosstalk among NOX enzymes, mitochondria, and the eNOS–ER–mitochondrial circuit can amplify redox dysfunction, while H_2_O_2_ transport, ROS-induced ROS release, iron-dependent chemistry, and nuclear/epigenetic signaling provide additional routes for localized signals to propagate. Loss of redox compartmentalization promotes impaired vasoprotection, endothelial activation and barrier dysfunction, and metabolic or pro-remodeling programs that contribute to pulmonary vascular remodeling and PAH progression. Solid arrows denote relationships supported in PAH or pulmonary vascular/endothelial models, whereas dashed arrows denote mechanisms that remain emerging, inferred, or extrapolated in PAH. Upward (↑) and downward (↓) arrows indicate increases and decreases, respectively. Created in BioRender. Marciano, D. (2026). https://BioRender.com/0vfwdws.

## 5. Intracellular Sources and Amplifiers of Redox Dysregulation

### 5.1. NADPH Oxidases

NADPH oxidases are dedicated ROS-generating enzymes that transfer electrons from NADPH to molecular oxygen, making them major regulated sources of oxidant signaling in the pulmonary vasculature. Endothelial cells express several NOX isoforms, including NOX1, NOX2, NOX4, and NOX5 [17,86]. These isoforms differ in their regulation, subcellular localization, and predominant ROS product. NOX1, NOX2, and NOX5 primarily generate superoxide, whereas approximately 90% of the electron flux through isolated NOX4 yields H_2_O_2_ [88]. These differences determine how far an oxidant signal can spread, which molecular targets it reaches, and whether the resulting response is adaptive or pathogenic.

#### 5.1.1. Endothelial NOX1 in PAH

Evidence for a pathogenic endothelial NOX pathway in PAH is most consistent for NOX1 and rests on both patient-derived cells and experimental disease. PAECs isolated from patients with PAH show NOX1 oxidase assembly associated with Gremlin-1-dependent proliferation [12]. In mice exposed to SU5416 and hypoxia, most commonly known as the Sugen Hypoxia model for PAH, infusion of the selective NOX1 inhibitor NOXA1ds suppressed the hemodynamic changes characteristic of PAH, while lungs from patients with idiopathic PAH and transcriptomic analysis of hypoxic human pulmonary endothelial cells in the same study implicated NOX1 in endoplasmic reticulum stress signaling and endothelial proliferation [11]. NOX1 therefore satisfies more of the evidence criteria set out in Section 1 than any other endothelial oxidant source discussed here, with supporting observations in human PAH tissue, patient-derived cells, and experimental PH.

#### 5.1.2. The Oxygen Dependence of NOX4

Among the endothelial NOX isoforms, NOX4 is notable because its activity shows a distinct dependence on oxygen availability, with an apparent O_2_ Km of approximately 18% compared with about 2–3% for NOX2 [88]. This biochemical property suggests that NOX4-dependent ROS generation may remain responsive across a broader range of oxygen concentrations, although these measurements were made in a partially purified, membrane-free preparation and do not establish oxygen sensitivity in intact pulmonary endothelium. In settings such as left-to-right shunting [96], altered pulmonary arterial oxygen tension makes NOX4 an interesting candidate for oxygen-dependent redox signaling, although a direct causal relationship remains unestablished.

#### 5.1.3. Divergent Findings for NOX4

Evidence for NOX4 in pulmonary vascular disease is genuinely divergent rather than merely context dependent, and this literature is better introduced by stating the conflict than by resolving it prematurely. On one side, NOX4 is the NADPH oxidase isoform most consistently upregulated by chronic hypoxia in mouse lung, localizes to the media of small pulmonary arteries, supports PASMC proliferation, and is increased approximately 2.5-fold in the vessel media of lungs from patients with idiopathic PAH [13]. In contrast, endothelial NOX4-derived H_2_O_2_ supports shear-dependent SHP2 sulfenylation and eNOS activation under physiologic conditions [15], and inducible endothelial Nox4 deletion produces vascular inflammation, medial hypertrophy, and endothelial dysfunction through loss of eNOS expression and NRF2-dependent heme oxygenase-1 signaling [16].

Several factors may explain this divergence. Cell type is the most apparent since the pathogenic observations largely derive from the arterial media and smooth muscle, while the protective observations derive from the endothelium [13,15,16]. Subcellular localization is a second factor because NOX4 has been assigned to the endoplasmic reticulum, perinuclear space, and nucleus, where it would encounter different targets [7,42,86]. Oxygen tension is a third factor given the enzyme’s unusually high apparent O_2_ Km and the wide range of oxygen tensions across the pulmonary circulation [88]. The reactive species itself is a fourth factor since H_2_O_2_ released by NOX4 differs from NOX1- and NOX2-derived superoxide in diffusion distance, protein targets, and reactivity toward nitric oxide [88]. Disease stage is a fifth as antioxidant treatment is effective early in chronic hypoxia and substantially weaker when started later [104]. The experimental model is a sixth since inhibitor studies in chronic hypoxia and in SU5416/hypoxia have produced different patterns of pulmonary vascular and right ventricular benefit [11,14].

The therapeutic implication of this section is therefore not that NADPH oxidase activity should be uniformly modulated, but instead that NOX1 and NOX4 represent different drug targets in different cells and compartments and that isoform-nonselective inhibition risks removing an adaptive endothelial signal along with a pathogenic one.

### 5.2. Mitochondrial Dysfunction

Mitochondria integrate oxygen sensing with metabolism, apoptosis, calcium homeostasis, and redox signaling in the pulmonary vasculature [93]. PAH is associated with altered substrate utilization and mitochondrial dynamics [105,106], although the resulting redox phenotype varies by cell type and disease context. Some of the classic mechanistic evidence comes from PASMCs, where attenuation of superoxide dismutase 2 (SOD2) alters mitochondrial redox signaling, promotes mitochondrial hyperpolarization, and contributes to apoptosis resistance [23]. Direct endothelial evidence includes increased mitochondrial superoxide and H_2_O_2_ during hypoxia in human PAECs [19] and impaired mitochondrial recovery following hypoxia–reoxygenation in BMPR2-deficient endothelial cells [18].

The species of mitochondrial oxidant matters as much as its quantity. In mice, mitochondrial-targeted catalase overexpression attenuated hypoxia-induced pulmonary hypertension and suppressed hypoxia-associated induction of NOX2 and NOX4, whereas SOD2 overexpression exacerbated the same responses [19]. Lowering mitochondrial H_2_O_2_ and lowering mitochondrial superoxide therefore produced opposite outcomes within a single model, one of the clearest demonstrations that reducing oxidant burden is not inherently protective and that the relevant reactive species must be specified.

Mitochondrial oxidants also participate in bidirectional signaling with other redox compartments. NOX-derived ROS can increase mitochondrial ROS production, while mitochondrial oxidants can activate NOX-dependent pathways. However, this reciprocal relationship has been characterized largely in systemic vascular and non-vascular systems [66]. Complex III-derived ROS participate directly in hypoxic signaling [21], and eNOS uncoupling has been linked to ER stress and mitochondrial dysfunction in 2,4-diamino-6-hydroxypyrimidine (DAHP)- and hypoxia-induced rodent PH models [25]. Interventions targeting eNOS coupling, ER stress, or mitochondrial ROS each interrupted this circuit and improved pulmonary vascular remodeling, hemodynamics, and nitric oxide bioavailability [25]. These findings position mitochondria as both a source and an amplifier of redox signaling and are considered further in Section 6.

### 5.3. eNOS Uncoupling and Nitrosative Stress

Loss of nitric oxide bioavailability is a defining feature of endothelial dysfunction in PAH and can result from both reduced nitric oxide production and altered eNOS catalytic activity. Coupled eNOS requires L-arginine and tetrahydrobiopterin (BH4), together with Ca^2+^/calmodulin-dependent electron transfer, to generate nitric oxide. BH4 deficiency, substrate limitation, S-glutathionylation, and inhibitory phosphorylation can all promote eNOS uncoupling [29,30,31]. Protein kinase C-dependent phosphorylation of eNOS at Thr495 disrupts calmodulin-dependent activity, reduces nitric oxide production, and increases eNOS-derived superoxide in human lung microvascular endothelial cells [28], while increased Thr495 phosphorylation in human PAH lung tissue supports the relevance of this mechanism to human disease [24].

When eNOS becomes uncoupled, protective nitric oxide signaling is lost alongside superoxide generation within the same endothelial microdomain. Superoxide rapidly reacts with nitric oxide to form peroxynitrite, further reducing nitric oxide bioavailability while increasing nitrosative stress. In pulmonary endothelial cells, nitrotyrosine formation is associated with impaired angiogenesis and eNOS uncoupling [35]. Several specific nitration targets provide a mechanistic link between nitrosative stress and pulmonary vascular dysfunction, including protein kinase G-1, whose nitration is increased in IPAH lungs and impairs kinase activity [32]. Nitration of prostacyclin synthase also increases, alongside reduced enzyme activity and prostacyclin production, in PAECs from a fetal lamb model of persistent pulmonary hypertension of the newborn [33]. Caveolar peroxynitrite additionally targets caveolin-1, reducing TRPV4 channel activity and increasing pulmonary arterial pressure [34]. Together, these findings show how localized nitrosative chemistry can disrupt several vasoprotective pathways without requiring a large change in global cellular ROS.

### 5.4. Labile Iron and Fenton Chemistry

Iron influences the biological consequences of peroxide signaling because redox-active ferrous iron converts H_2_O_2_ to highly reactive hydroxyl radical through Fenton chemistry [37]. Ferritin storage, mitochondrial iron handling, and regulation of the labile iron pool influence whether locally generated H_2_O_2_ remains a signaling intermediate or enters more reactive radical chemistry. This distinction matters for compartmentalized redox signaling because H_2_O_2_ and redox-active iron must converge in the same local environment for Fenton chemistry to occur.

Systemic iron status and intracellular labile iron should be kept separate. Iron deficiency occurs in roughly 40% of patients with idiopathic PAH and is associated with reduced exercise capacity independent of anemia [107]. Circulating iron status nonetheless does not reflect endothelial redox-active iron [108] since the labile iron pool is regulated locally and can vary independently of total cellular or systemic iron [37]. This creates an apparent contradiction: iron deficiency is common and clinically adverse at the systemic level, while intracellular labile iron is proposed to drive endothelial injury in the same disease. The two are not inconsistent because a local pool can rise while systemic stores fall. However, they represent biologically distinct therapeutic problems, and neither has been shown to modify the other.

### 5.5. Ferroptosis

Iron-dependent oxidative chemistry and ferroptosis are related but not equivalent, and the distinction matters for both mechanism and therapy. Fenton chemistry occurs when redox-active Fe^2+^ converts H_2_O_2_ to hydroxyl radical wherever the two converge [37], producing indiscriminate oxidative damage rather than a regulated cell death program. Ferroptosis is a regulated form of cell death defined by iron-dependent peroxidation of membrane phospholipids that proceeds when glutathione peroxidase 4 (GPX4) and related phospholipid repair systems are overwhelmed [40,41]. A cell can sustain substantial Fenton-driven oxidative injury without undergoing ferroptosis, and ferroptosis can be pharmacologically inhibited without altering bulk iron-dependent oxidation.

The sequence relevant to PAH therefore runs from accumulation of the intracellular labile iron pool, through Fenton-mediated initiation of lipid radical chemistry, to propagation of phospholipid peroxidation in membranes, and, if GPX4 and related defenses fail, to ferroptosis, although evidence is uneven across these steps. In monocrotaline-induced PAH, rat PAECs show an increased labile iron pool, increased lipid peroxidation, downregulation of GPX4 and ferritin heavy chain 1, and upregulation of NOX4, all of which the ferroptosis inhibitor ferrostatin-1 blocked [36]. Ferroptosis inhibition reduces endothelial injury and pulmonary vascular remodeling in monocrotaline rats [36,38] and SU5416/hypoxia mice [39]. Fenton chemistry itself has not been demonstrated directly in pulmonary endothelium and is inferred from the underlying chemistry [37].

Human evidence remains indirect, with ferroptotic human PAECs altering PASMC and monocyte phenotypes in vitro and analysis of the Vanderbilt BioVU repository linking single-nucleotide polymorphisms in six ferroptosis-related genes to greater pulmonary hypertension severity [38]. These findings support the potential relevance of ferroptosis to human disease but do not establish it as a dominant endothelial mechanism or as a validated therapeutic target in PAH.

Iron therefore enters PAH endothelial biology in two distinct ways: as a systemic deficiency with established clinical associations and as a local pool that determines whether H_2_O_2_ remains a signal or initiates membrane peroxidation. Evidence that the second contributes causally to human disease remains experimental.

### 5.6. Additional Enzymatic and Organelle ROS Sources

Additional enzymatic and organelle pathways can influence endothelial redox tone but are less well established in PAH. Xanthine oxidoreductase generates superoxide and H_2_O_2_ and is activated by moderate hypoxia in arterial endothelial cells [70]. Peroxisomes, cytochrome P450 epoxygenases, ER oxidative protein folding, and lipoxygenases provide additional sources or regulators of endothelial oxidant signaling [71,72,73,74,75]. Because most evidence for these pathways is extrapolated from broader endothelial or non-endothelial systems, they should be viewed as candidate contributors whose support remains weaker than that for NOX-dependent signaling, mitochondrial dysfunction, or eNOS uncoupling.

## 6. Intercompartmental Redox Crosstalk

The preceding section described oxidant sources individually, but their separation is the exception rather than the rule. Compartmentalization is maintained not by physical isolation but by the balance between transport and local scavenging, and the same connections that allow a localized signal to reach its targets also allow a dysregulated one to propagate. Four forms of crosstalk are relevant to PAH and are considered below (Figure 2). Taken together, they explain why loss of compartmentalization is progressive rather than discrete because a change confined to one compartment does not remain confined, and the resulting phenotype reflects the connections between compartments as much as the activity of any single source.

### 6.1. NADPH Oxidase–Mitochondrial Crosstalk

NOX-derived oxidants and mitochondrial ROS production are mutually reinforcing, so a signal originating at the plasma membrane can become a mitochondrial one without changing global cellular ROS [66]. The pulmonary circulation provides evidence for this coupling in both directions. Reducing mitochondrial H_2_O_2_ with mitochondria-targeted catalase lowered lung NOX2 and NOX4 expression and attenuated hypoxia-induced PH in mice, demonstrating that a mitochondrial intervention can suppress membrane oxidase expression [19]. Conversely, ferroptotic rat PAECs showed upregulation of NOX4 alongside mitochondrial lipid peroxidation [36]. The reciprocal relationship between NOX enzymes and mitochondria has been characterized most fully in other lung diseases and in non-pulmonary systems [66,67] and should be regarded as extrapolated wherever direct pulmonary endothelial evidence is absent.

### 6.2. ROS-Induced ROS Release

Mitochondrial oxidant release can also propagate between mitochondria through ROS-induced ROS release, in which opening of the mitochondrial permeability transition pore or the inner membrane anion channel produces a regenerative cycle in which oxidant release from one mitochondrion triggers release from its neighbors, converting a local event into a cell-wide event [68]. Brief openings appear to serve an adaptive housekeeping function, whereas sustained openings can propagate destructively from mitochondrion to mitochondrion [68]. This mechanism is well characterized in cardiomyocytes and isolated mitochondria but has not been demonstrated in pulmonary endothelial cells in PAH.

### 6.3. The eNOS–Endoplasmic Reticulum–Mitochondrial Circuit

The strongest experimental evidence that pulmonary vascular redox compartments behave as a connected circuit comes from studies of eNOS uncoupling. In DAHP- and hypoxia-induced PH, eNOS uncoupling, ER stress, and mitochondrial dysfunction reinforced one another, and folic acid, phenylbutyric acid, and MitoTempo, three agents directed at three different compartments, each improved pulmonary hemodynamics and remodeling while preserving nitric oxide bioavailability [25]. The central observation is that intervention at any one node affects the whole and that these compartments do not merely coexist but behave as a single circuit. Because this evidence comes from experimental PH rather than human disease, the circuit should be regarded as demonstrated in models and hypothesized in patients.

### 6.4. H_2_O_2_ Transport and Buffering as the Coupling Medium

What connects these compartments is not diffusion of a generic oxidant but H_2_O_2_ flux through defined channels against a defined local scavenging capacity. Aquaporins facilitate H_2_O_2_ movement across membranes [64,65], while peroxiredoxins, glutathione peroxidases, and catalase determine how far a peroxide signal travels before it is quenched [85]. The ER illustrates this principle directly since GPx8 prevents leakage of H_2_O_2_ generated during oxidative protein folding into the cytosol [73]. A fall in local buffering capacity therefore extends the reach of an otherwise unchanged signal, and the same anatomical connections that support physiologic compartmentalization become the route by which it is lost. This transport-and-buffering view is established largely in non-pulmonary systems and is applied here by extrapolation.

This crosstalk has direct therapeutic consequences and may explain the modest results of single-compartment interventions. If NOX-derived and mitochondrial oxidants amplify one another, inhibiting either source alone may be buffered by the other, which is consistent with the partial pulmonary vascular effects of both NOX inhibition [14] and mitochondrial antioxidant treatment [20] in experimental PH. Conversely, the eNOS–ER–mitochondrial studies showed improvement from agents acting at any of the three nodes [25], indicating that some circuits can be interrupted at more than one point. Which interventions require combined targeting, and which can be addressed at a single node, remains undetermined in pulmonary endothelium, and this framework raises a concrete experimental question.

## 7. Endothelial Consequences and Redox-Responsive Programs

### 7.1. Loss of Nitric Oxide Signaling

A major consequence of endothelial redox dysregulation in PAH is loss of nitric oxide bioavailability, which affects far more than acute vasodilation. Once generated by the endothelium, nitric oxide readily diffuses into adjacent pulmonary arterial smooth muscle cells, where it activates soluble guanylate cyclase and increases cGMP signaling. This pathway promotes relaxation while also restraining smooth muscle proliferation and vascular remodeling [6,24]. In PAH, reduced nitric oxide production is compounded by eNOS uncoupling and rapid consumption of nitric oxide by superoxide, weakening this endothelial-to-smooth muscle signaling axis and shifting the pulmonary vasculature toward vasoconstriction and remodeling [29,30,34].

Nitric oxide also acts at the endothelial–blood interface, where it limits platelet activation and leukocyte adhesion [6,24]. This antiplatelet effect persists under flow, positioning endothelial nitric oxide as an important regulator of platelet–vessel wall interactions in the circulation [109]. Loss of nitric oxide promotes a more adhesive and prothrombotic luminal surface that can amplify inflammatory and vasoactive signaling within the vascular wall. Together, these effects extend the consequences of redox stress beyond vascular tone and provide another route through which nitric oxide deficiency can reinforce pulmonary vascular remodeling.

### 7.2. Inflammatory Activation and Barrier Dysfunction

Inflammatory activation and barrier dysfunction are closely linked consequences of endothelial redox dysregulation because oxidant-sensitive pathways regulate both leukocyte recruitment and cytoskeletal organization. Activation of NF-κB and AP-1 increases adhesion molecule and cytokine expression [101], while Nck recruitment to PECAM-1 provides a defined mechanism linking oxidative stress to canonical NF-κB signaling [102]. In the inflammatory environment of PAH, ROS and reactive nitrogen species generated by endothelial and perivascular immune cells can reinforce these pathways [99,100], promoting a feed-forward cycle of endothelial activation and leukocyte recruitment.

Barrier regulation adds another layer because the effect of an oxidant signal depends on the molecular target it engages. TNIK promotes TNF-dependent phosphorylation of ezrin/radixin/moesin proteins, cytoskeletal remodeling, and endothelial gap formation, yet oxidation of a single cysteine reversibly inhibits the kinase [103]. This target-specific behavior illustrates how individual redox signals can restrain one pathway while the broader inflammatory environment still favors permeability and loss of endothelial integrity. Loss of barrier integrity exposes subendothelial compartments to plasma proteins and circulating mediators that normally remain largely intravascular. Increased permeability also facilitates leukocyte trafficking and paracrine communication with surrounding vascular cells, reinforcing inflammation and remodeling.

### 7.3. Metabolic Reprogramming

Metabolic reprogramming is tightly integrated with endothelial redox state in PAH rather than representing a separate downstream abnormality. Pulmonary vascular cells shift toward glycolysis and altered mitochondrial glucose oxidation [105,110], changes that can support proliferation and survival under stress. In endothelium, BMPR2 mutations create abnormal metabolic requirements, including greater glutamine dependence [78,79], while endothelial regeneration depends on coordinated changes in glycolytic and mitochondrial metabolism [111]. These changes alter mitochondrial electron flow and the reducing equivalents available to maintain redox homeostasis.

Pulmonary overcirculation provides direct endothelial evidence linking mitochondrial dynamics to this metabolic shift. In PAECs isolated from shunt lambs, nitration-dependent activation of the small GTPase RhoA drives mitochondrial fission together with increased mitochondrial ROS, HIF-1α signaling, and glycolysis [112]. Inhibiting the fission protein Drp1 or restoring fusion reduces glycolysis and improves mitochondrial function, indicating that altered mitochondrial morphology can actively drive metabolic reprogramming rather than simply accompany it [112].

ROS can reshape endothelial metabolism by affecting HIF signaling, mitochondrial function, and redox-sensitive metabolic enzymes [105,110]. Metabolic reprogramming can then feed back on redox state by changing mitochondrial electron flow and NADPH availability for NOX activity and antioxidant defense [86,105,110]. Metabolic state also influences endothelial gene expression by changing the availability of metabolites required for chromatin regulation. In a BMPR2-Notch1 human endothelial–smooth muscle co-culture model, glycolytic metabolism increased acetyl-CoA availability and p300-mediated histone acetylation at enhancers required for endothelial proliferation and homeostasis [111]. These interactions can stabilize a persistent redox, metabolic, and transcriptional phenotype that reinforces endothelial dysfunction.

### 7.4. Nuclear Redox Signaling and Epigenetic Remodeling

#### 7.4.1. Mechanisms Established in Pulmonary Vascular Disease

Three observations link redox state to endothelial chromatin regulation with reasonably secure evidence. Epigenetic attenuation of SOD2 provides a pulmonary vascular precedent, although it was established largely in smooth muscle rather than endothelium [23]. Endothelial cells from patients with PAH exhibit remodeled active enhancer landscapes [50], which is direct human evidence that endothelial transcriptional regulation is altered in the disease, even though the study does not attribute this remodeling to a defined oxidant source. Shear stress reshapes the endothelial epigenome through KLF4-dependent chromatin remodeling [51], p300-dependent NOS3 transcription [59], and flow-dependent DNA methylation [60,61], and this mechanism is well established in endothelial biology but has been defined in systemic rather than pulmonary models.

#### 7.4.2. Proposed Mechanisms Not Yet Established in PAH

The mechanisms described in this subsection are biochemically plausible and in several cases well characterized outside the pulmonary circulation, but none has been shown to operate in pulmonary endothelial cells in PAH. We present them as hypotheses generated by the compartmentalized redox framework and testable within it, not as established components of PAH pathobiology.

Nuclear redox signaling offers a potential mechanism through which localized oxidant signals could produce longer-lasting changes in endothelial gene expression. Oxidatively modified DNA can recruit chromatin regulatory complexes in non-PAH systems [52], and endothelial PPAR-γ-UBR5/ATMIN signaling links DNA repair to endothelial homeostasis [113]. As noted in Section 3.2, nuclear NOX4 has been reported in human umbilical vein endothelial cells [42] but not demonstrated in pulmonary endothelium. Together, these observations connect redox state, DNA repair, and chromatin regulation but have not yet identified a defined oxidant-driven nuclear pathway in PAH endothelium.

Among oxidative DNA modifications, 8-oxoguanine (8-oxoG) is mechanistically interesting because it can alter base pairing and influence transcription by recruiting OGG1 and other base excision repair proteins [43]. This provides a potential route through which localized DNA oxidation could influence gene expression rather than functioning only as a marker of injury. Its steady-state abundance reflects formation, repair, and further oxidation to secondary guanine products [44,45,46], so changes in 8-oxoG cannot be interpreted simply as cumulative oxidant exposure. This also affects the interpretation of circulating 8-hydroxy-2′-deoxyguanosine as a biomarker (Section 8.1) since a measured change in that species reflects the balance of formation, excision, and further oxidation rather than oxidant exposure alone. Whether locus-specific 8-oxoG and OGG1 signaling contribute to PAH endothelial transcription remains unresolved.

Redox and metabolic conditions may also influence Fe^2+^- and α-ketoglutarate-dependent chromatin enzymes, including the TET proteins that generate 5-hydroxymethylcytosine [47,48] and the JmjC-domain histone demethylases [49]. Their dependence on reduced iron and metabolic cofactors creates a direct biochemical interface between redox state and chromatin regulation. Direct coupling of these changes to a defined ROS source has not been established in human PAH, so causal studies will need to link oxidant source, locus-specific chromatin change, transcriptional output, and endothelial phenotype within the same system.

Nuclear redox signaling therefore offers a plausible route from a transient oxidant signal to a durable endothelial phenotype, which is why it merits attention. At present, however, it is a framework for designing experiments in PAH rather than a description of established disease mechanisms, and Figure 3 distinguishes these proposed mechanisms from those with direct experimental support.

## 8. Circulating Markers of Redox Dysregulation and Endothelial Dysfunction in PAH

Circulating biomarkers provide an accessible window into PAH redox biology, although few are specific to pulmonary endothelium. These markers are also not interchangeable, and grouping them as indices of oxidative stress obscures what each one measures. F2-isoprostanes and 8-hydroxy-2′-deoxyguanosine are products of oxidant chemistry acting on lipids and DNA, respectively. Nitrotyrosine reports peroxynitrite-mediated protein modification. Asymmetric dimethylarginine (ADMA) is a competitive inhibitor of nitric oxide synthase rather than an oxidation product, and circulating endothelial extracellular vesicles report endothelial activation and injury rather than oxidation. None is specific to the pulmonary endothelium, and none identifies the oxidant source or subcellular compartment from which a signal arises. Table 2 classifies these markers by the process they measure, their cellular and pulmonary specificity, their mechanistic linkage to a defined redox source, the strength of prognostic evidence in PAH, and the extent of clinical validation. The mechanistic linkage column is particularly informative because no currently available circulating marker is anchored to a compartment-specific redox pathway.

### 8.1. Stable Products of Oxidant Chemistry

F2-isoprostanes are among the best-supported circulating markers of oxidant injury in PAH. Urinary F2-isoprostanes measured at diagnostic right heart catheterization independently predicted 3-year all-cause mortality in 110 patients with incident PAH and were the only biomarker in that study to remain independently associated with mortality after adjustment for patient characteristics [114]. In a separate cohort of 80 patients with idiopathic PAH, plasma 15-F2t-isoprostane was elevated and independently predicted mortality while also tracking disease severity [115]. The same study detected increased 15-F2t-isoprostane within remodeled pulmonary vessels, further supporting the biological relevance of this oxidation product within diseased pulmonary vasculature. Together, these findings support isoprostanes as stable markers of oxidant burden and prognosis, although they do not identify the endothelial source or the subcellular compartment where oxidation occurs.

Other circulating oxidation products have been studied less extensively, and their supporting data are weaker. Plasma nitrotyrosine fell after recombinant human angiotensin-converting enzyme 2 in a small phase IIa study [116], where it functioned as a pharmacodynamic readout rather than as a validated marker. Serum 8-hydroxy-2′-deoxyguanosine correlated with worse functional class and shorter 6-minute walk distance in a 25-patient cohort but did not differ significantly between patients and healthy controls [117], limiting its value as a diagnostic or disease-defining measure. Because neither measurement identifies a pulmonary endothelial source, these findings remain hypothesis generating rather than sufficient for routine clinical use.

### 8.2. Circulating Markers of Nitric Oxide Dysfunction

ADMA is not an oxidation product but provides a circulating measure of impaired nitric oxide biology through competitive inhibition of nitric oxide synthase. Plasma ADMA was elevated in idiopathic PAH and independently predicted mortality in a 57-patient cohort [118]. In a prospective study of 215 patients, reductions in ADMA during therapy were associated with better 3-year and 5-year survival and added prognostic information to N-terminal pro-brain natriuretic peptide [119]. Experimental studies in PAECs provide a mechanistic endothelial link by showing that ADMA promotes eNOS uncoupling, mitochondrial protein nitration, and mitochondrial ROS [120]. Because systemic methylarginine metabolism and renal handling also influence circulating ADMA, it is best interpreted as a marker of nitric oxide pathway dysfunction rather than as a direct measure of pulmonary endothelial ROS.

### 8.3. Circulating Extracellular Vesicles with Endothelial Markers

Earlier PAH studies used the terms microparticle or microvesicle, whereas the MISEV2023 guidelines recommend the generic term extracellular vesicle (EV) with operational descriptors unless biogenesis is established [121]. Circulating PECAM-positive and VE-cadherin-positive particles were increased in precapillary pulmonary hypertension and associated with hemodynamic severity [122], although these populations cannot be assigned specifically to pulmonary endothelium. Other PAH cohorts identified increased procoagulant and endoglin-positive particles [123], as well as higher levels of EV populations classified by endothelial surface markers, together with fewer circulating progenitor cells [124]. Persistence of this vesicle-to-progenitor cell imbalance after therapy suggests ongoing endothelial injury and impaired repair rather than a direct measure of oxidative stress.

Experimental studies further suggest that redox signaling can influence both EV release and biological activity. H_2_O_2_ alters the subtype distribution of particles released by human pulmonary microvascular endothelial cells [125], while EV preparations from monocrotaline-treated animals can transmit pulmonary hypertensive changes to recipient mice [126]. Human studies have not yet identified a circulating EV population with endothelial markers that maps to a defined PAH redox pathway, and translation will require standardized preanalytic handling, EV separation, characterization, and reproducible quantification [121]. Combining stable oxidation products with EV populations carrying endothelial markers may ultimately provide a more informative view of endothelial redox dysfunction than a single global oxidative stress assay.

Circulating markers therefore confirm that oxidant chemistry and endothelial injury increase in PAH and carry prognostic information, but none report where within the endothelial cell this chemistry occurs, which compartment-directed therapy would need to know.

**Table 2:** Classification of circulating markers proposed for redox phenotyping in PAH, by the biological process measured, cellular and pulmonary specificity, mechanistic linkage to a defined redox source, prognostic evidence and clinical validation.

Marker	Biological Process Measured	Cellular and Pulmonary Specificity	Mechanistic Link to a Redox Source	Prognostic Evidence in PAH	Clinical Validation
Urinary F2-isoprostanes	Non-enzymatic arachidonate peroxidation; cumulative lipid oxidation	None; whole-body	None; no defined oxidant source or compartment	Independently predicted 3-year mortality in 110 patients with incident PAH [114]	Strongest of these markers; not used prospectively to select therapy
Plasma 15-F2t-isoprostane	Lipid peroxidation, plasma pool	None; whole-body, though detected within remodeled vessels	None	Elevated in IPAH and independently predicted mortality in 80 patients [115]	None
Plasma nitrotyrosine	Peroxynitrite-mediated protein tyrosine nitration	None; all nitrated proteins, whole-body	Indirect; implies NO-superoxide convergence without specifying a source	Fell after recombinant human ACE2 in a phase IIa study [116]	None; pharmacodynamic readout only
Serum 8-OHdG	Oxidative DNA base damage, net of formation, excision and further oxidation [44,45,46]	None; any nucleated cell and the nucleotide pool	Weak; not attributable to a source or compartment	Correlated with functional class and 6MWD but did not differ from controls in 25 patients [117]	None
Plasma ADMA	Competitive NOS inhibition; methylarginine metabolism	None; systemic NO pathway, and not an oxidation product	Indirect; impairs NO production rather than reporting oxidation. Promotes eNOS uncoupling and mitochondrial ROS in PAECs [120]	Predicted mortality in 57 patients [118]; falling levels predicted survival in 215 patients and added to NT-proBNP [119]	Best characterized as a prognostic adjunct
Endothelial EVs (PECAM+, VE-cadherin+)	Endothelial activation, injury and vesicle release	Endothelial but not pulmonary-specific	Indirect; H_2_O_2_ alters EV subtype distribution in HPMVECs [125]	Correlate with hemodynamic severity [122,123,124]	None; assays not standardized [121]

Abbreviations: 6MWD, 6-minute walk distance; 8-OHdG, 8-hydroxy-2′-deoxyguanosine; ACE2, angiotensin-converting enzyme 2; ADMA, asymmetric dimethylarginine; eNOS, endothelial nitric oxide synthase; EV, extracellular vesicle; H_2_O_2_, hydrogen peroxide; HPMVEC, human pulmonary microvascular endothelial cell; IPAH, idiopathic pulmonary arterial hypertension; NO, nitric oxide; NOS, nitric oxide synthase; NT-proBNP, N-terminal pro-brain natriuretic peptide; PAECs, pulmonary arterial endothelial cells; PAH, pulmonary arterial hypertension; PECAM, platelet endothelial cell adhesion molecule; ROS, reactive oxygen species.

## 9. Therapeutic Implications

The therapeutic challenge in PAH is not simply to reduce oxidative stress, but to identify which redox pathways drive endothelial dysfunction and can be targeted without disrupting adaptive signaling. Established PAH therapies can influence endothelial redox balance indirectly through endothelin, NO-cGMP, prostacyclin, and activin/BMP signaling. Redox-directed strategies take a different approach by targeting defined sources or compartments, including NOX enzymes, mitochondrial ROS, eNOS uncoupling, and iron-dependent chemistry. Model context remains important because chronic hypoxia emphasizes oxygen sensing and muscularization, monocrotaline produces prominent endothelial injury and inflammation, and pulmonary artery banding models right ventricular pressure overload without a pulmonary vascular lesion. The assessment that follows therefore distinguishes whether an intervention prevented disease or reversed it once established, whether benefit was demonstrated in the pulmonary vasculature or only in the right ventricle, and whether target engagement was shown at the proposed redox step rather than inferred from a global ROS assay. Table 3 summarizes representative redox-directed studies in experimental PH and human PAH/PH on this basis.

### 9.1. Established PAH Therapies and Redox Signaling

Current PAH therapies target four major signaling pathways involving endothelin-1, nitric oxide-cGMP, prostacyclin, and activin/BMP signaling [127]. Although these therapies were not developed to directly modify redox state, several influence pathways that are closely coupled to endothelial oxidant production and nitric oxide bioavailability. In SU5416/hypoxia rats, bosentan reduced superoxide and H_2_O_2_ production, protein nitration, NADPH oxidase activation, and eNOS uncoupling [128]. Endothelin receptor blockade can thus reduce downstream oxidative and nitrosative stress, although comparable redox effects have not been established in patients.

Therapies targeting the NO-cGMP pathway act largely downstream of impaired nitric oxide bioavailability rather than correcting the oxidant processes responsible for its loss. In a small human PAH study, sildenafil reduced circulating 4-hydroxynonenal while improving pulmonary hemodynamics and functional capacity [129], suggesting that restoration of NO-cGMP signaling can also shift systemic markers of oxidative stress. Prostacyclin pathway therapies reinforce another vasoprotective signaling axis that nitration of prostacyclin synthase can compromise [33]. Sotatercept targets activin signaling and adds a distinct therapeutic axis in PAH, but whether it directly modifies endothelial redox signaling remains unknown [127].

One approach within this group is redox-defined rather than merely redox-adjacent. Soluble guanylate cyclase is a heme protein, and under oxidative stress its prosthetic heme is oxidized and lost, producing a nitric oxide-insensitive and degradation-prone form that neither endogenous nitric oxide nor sGC stimulators can activate [130]. sGC activators bind the vacant heme pocket and selectively activate this oxidized form, so they act preferentially in oxidatively stressed vessels [130]. In the open-label phase 1b ATMOS trial, single inhaled doses of the sGC activator mosliciguat produced mean peak reductions in pulmonary vascular resistance ranging from 21% to 38% across a 0.24–4.0 mg dose range in 38 participants with PAH or chronic thromboembolic pulmonary hypertension, including nitric oxide-non-responsive participants with baseline pulmonary vascular resistance of 5 Wood units or greater, with effects sustained over a 3-h observation period and no safety-relevant change in systemic vascular resistance [131]. This is early, uncontrolled, single-dose evidence, but it is notable because the mechanism is defined by the target’s oxidation state rather than oxidant abundance. Established therapies may thus improve redox balance as a downstream consequence of correcting disease-relevant signaling, whereas redox-directed therapies are designed to act on a defined oxidant source, reactive species, subcellular compartment, or redox-modified target.

### 9.2. Targeting NADPH Oxidases

NOX inhibition offers a source-targeted strategy, but its therapeutic value depends on isoform selectivity, as discussed in Section 5.1.3. PAECs from patients with PAH show pathogenic NOX1 assembly [12], whereas endothelial NOX4 can support adaptive H_2_O_2_ signaling under physiologic shear [15] and its deletion produces endothelial dysfunction [16]. In mice given GKT137831 during the final 10 days of a 3-week hypoxic exposure, the inhibitor attenuated right ventricular hypertrophy and pulmonary artery wall thickness but did not attenuate the rise in right ventricular systolic pressure or the muscularization of small arterioles [14]. Selective NOX1 inhibition with NOXA1ds produced broader hemodynamic improvements in SU5416/hypoxia and suppressed endothelial NOX1-dependent ER stress signaling, with supporting observations in lungs from patients with idiopathic PAH [11]. These studies support greater mechanistic precision through isoform-selective approaches that suppress pathogenic NOX signaling without broadly interfering with adaptive redox pathways.

### 9.3. Targeting Mitochondrial Redox Dysfunction and Metabolism

The MitoQ data illustrate why pulmonary vascular and right ventricular effects must be reported separately. In isolated perfused mouse lungs, MitoQ blunted acute hypoxic pulmonary vasoconstriction. In mice exposed to 10% O2 for four weeks, however, MitoQ did not affect the development of chronic hypoxia-induced pulmonary hypertension, but it attenuated right ventricular remodeling after both chronic hypoxia and pulmonary artery banding, a model with no pulmonary vascular lesion [20]. The same study found that ROS decreased in PASMCs but increased in the right ventricle during chronic hypoxia, which is consistent with the benefit arising in the right ventricle rather than in the pulmonary vasculature [20]. A mitochondria-directed antioxidant that improves right ventricular function is clinically valuable, but MitoQ should not be presented as evidence that mitochondrial oxidants drive pulmonary vascular remodeling. Available mitochondria-directed interventions are also not endothelial-selective. MitoQ, MitoTEMPO, and TEMPOL are research compounds that are not approved therapies for pulmonary hypertension and have not been evaluated in controlled PAH trials.

Metabolic targeting addresses a closely linked component of the redox-mitochondrial phenotype. Dichloroacetate (DCA) reversed established monocrotaline PH in rats [132] and reduced mean pulmonary artery pressure and pulmonary vascular resistance in a subset of patients with idiopathic PAH [133]. Responses in the small open-label human study were heterogeneous and associated with SIRT3 and UCP2 genotype, suggesting that baseline redox-metabolic state may influence therapeutic response.

### 9.4. Restoring eNOS Coupling and Nitric Oxide Signaling

Restoring eNOS coupling directly targets the convergence of reduced nitric oxide production and increased endothelial superoxide. BH4 deficiency promotes PH, while augmenting endothelial BH4 synthesis is protective [26]. Pharmacologic restoration of BH4 availability or recycling improves eNOS coupling across rodent and newborn pig models [134,135,136]. Dihydrofolate reductase (DHFR) supports BH4 recycling and provides another approach to maintaining eNOS coupling. Endothelial-specific DHFR expression prevents disease, while DHFR plasmid delivery after disease induction reverses established PH [27], making this one of the few interventions in this literature demonstrated in a reversal rather than a prevention design.

Recent work in DAHP- and hypoxia-induced PH models linked eNOS uncoupling to ER stress and mitochondrial dysfunction, defining an interconnected redox–stress circuit [25]. Folic acid, phenylbutyric acid, and MitoTempo improved pulmonary hemodynamics and remodeling while targeting eNOS coupling, ER stress, or mitochondrial ROS at different points within this pathway [25], and this study also provides the strongest available demonstration of target engagement at the proposed redox step.

Human evidence for direct eNOS recoupling remains limited despite the strength of the experimental data. Oral sapropterin was well tolerated in a small open-label study of PAH and inoperable chronic thromboembolic pulmonary hypertension, with a signal for improved 6-minute walk distance at intermediate dosing but without significant change in measured nitric oxide synthesis or oxidant stress markers [137]. Dietary nitrate provides a complementary route to nitric oxide generation and reverses established hypoxic PH in mice through pathways partly dependent on eNOS and xanthine oxidoreductase [69]. Together, these studies show that nitric oxide biology can be restored through several mechanistic routes while also underscoring the gap between experimental efficacy and clinical validation.

### 9.5. NRF2 Activation and the Limits of Broad Antioxidant Therapy

Broad antioxidant studies illustrate the limits of lowering oxidant tone without targeting a defined source or compartment. TEMPOL reduced right ventricular systolic pressure in chronic hypoxia and SU5416/hypoxia rats but did not correct remodeling, and medial hypertrophy and adventitial fibrotic lesions were more pronounced after TEMPOL treatment in the severe model [138]. N-acetylcysteine was most effective when given early during chronic hypoxia and substantially weaker when treatment began later [104], consistent with a diminishing effect once structural remodeling is established. These results are not simply negative because an intervention that lowers oxidant tone while worsening remodeling suggests that some of the oxidant signal being removed was adaptive, supporting compartment-directed rather than global approaches.

NRF2 activation has progressed to clinical testing, although efficacy in PAH remains unproven. Initial results from the small phase 2 LARIAT PAH cohort showed an early placebo-corrected improvement in 6-minute walk distance with bardoxolone methyl [139]. The phase 3 CATALYST trial in connective tissue disease-associated PAH was terminated during the COVID-19 pandemic before confirmatory efficacy could be established [140].

Vitamin C further illustrates the context dependence of antioxidant therapy. Severe deficiency can cause reversible pulmonary hypertension [141], whereas pharmacologic ascorbate can become pro-oxidant through extracellular H_2_O_2_ generation and iron-dependent Fenton chemistry [142,143]. The same compound can therefore have opposite redox effects depending on dose, compartment, and iron availability.

### 9.6. Iron, Ferroptosis and Redox-Active Metal Chemistry

The therapeutic implications of iron depend on distinguishing systemic iron deficiency from local redox-active iron chemistry, as set out in Section 5.4 and Section 5.5. Two randomized crossover trials using intravenous ferric carboxymaltose or iron dextran corrected iron deficiency in idiopathic or heritable PAH without improving exercise capacity or cardiopulmonary hemodynamics at 12 weeks [108]. By contrast, ferrostatin-1 improved pulmonary vascular endpoints in monocrotaline studies [36,38], and ferroptosis suppression was also beneficial in SU5416/hypoxia [39]. These interventions target different problems, and neither has been shown to affect the other. Human evidence for endothelial ferroptosis remains associative, so translation will require defining the relevant labile iron and lipid peroxide state, demonstrating that it can be modified in patients, and showing that doing so does not worsen systemic iron deficiency.

### 9.7. Translational Priorities for Redox-Directed Therapy

Redox-directed therapy should move beyond nonspecific antioxidant approaches toward interventions defined by oxidant source, subcellular compartment, and molecular target. The relevant reactive species and endothelial consequence should be demonstrated experimentally, with target engagement measured at the proposed redox step rather than inferred from a global ROS assay.

A limitation running through this literature is that most redox-directed interventions in experimental pulmonary hypertension have been administered before or concurrently with the initiating stimulus and therefore test prevention rather than reversal of established disease. Dichloroacetate reversed established monocrotaline PH [132], dietary nitrate reversed established hypoxic PH [69], and endothelial DHFR delivery reversed disease after induction [27]. Conversely, N-acetylcysteine was substantially more effective in the initial phase of chronic hypoxia than later [104], which is the pattern expected if redox dysregulation initiates remodeling that subsequently becomes redox independent. Because patients present with established disease, prevention studies should not be read as supporting therapeutic claims, and Table 3 records this distinction.

Clinical translation will also require evidence that modifying the selected redox pathway improves pulmonary hemodynamics or clinical outcomes without disrupting adaptive redox signaling. Biomarkers may help identify the relevant redox phenotype and explain heterogeneous responses, as illustrated by SIRT3/UCP2-associated variation with DCA [133]. For endothelial redox therapy, target engagement should confirm modification of the intended oxidant source or subcellular compartment rather than rely on a nonspecific oxidative biomarker. The central therapeutic opportunity is not broad suppression of oxidants but selective correction of pathogenic redox signaling at the source and compartment where it contributes to disease.

Although no redox-directed therapy has yet been shown to modify pulmonary vascular disease in patients, the interventions with the clearest mechanistic definition are the least tested in humans, while those tested in humans have the least compartment specificity.

**Table 3:** Representative therapeutic studies informing redox-directed strategies in experimental pulmonary hypertension and human PAH/PH, classified by treatment timing, pulmonary vascular outcome, right ventricular outcome, endothelial target engagement, and extent of human validation.

Intervention	Model or Study	Timing	Pulmonary Vascular Outcome	RV Outcome	Endothelial Target Engagement	Human Validation
Selective NOX1 inhibition (NOXA1ds)	Mouse SU5416/hypoxia [11]	Concurrent with disease induction	Improved PAH-associated hemodynamics	Not separately reported	Yes; endothelial NOX1-PERK-eIF2α-ATF4 signaling, with supporting human IPAH lung data	None; preclinical with supportive human tissue
NOX1/4 inhibition (GKT137831)	Mouse chronic hypoxia [14]	Delayed; final 10 days of a 3-week exposure	Reduced PA wall thickness; did not attenuate the rise in RVSP or small-arteriole muscularization	Reduced RV hypertrophy	Not isoform selective; effects seen in both HPAECs and HPASMCs	None
Mitochondria-targeted antioxidant (MitoQ)	Mouse chronic hypoxia and pulmonary artery banding [20]	Concurrent with disease induction	Did not prevent chronic hypoxia-induced PH; blunted acute HPV in isolated lungs	Attenuated RV remodeling after both chronic hypoxia and PA banding	Not demonstrated; not endothelial selective	None
Mitochondria-targeted catalase vs SOD2 overexpression	Transgenic mice, chronic hypoxia [19]	Genetic; present throughout	Catalase attenuated PH and vessel muscularization; SOD2 overexpression exacerbated both	Not separately reported	Partial; reduced lung NOX2 and NOX4 expression	None
PDK inhibition (DCA)	Rat monocrotaline PH; open-label idiopathic PAH study [132,133]	Reversal of established disease in rats	Reversed established PH in rats; reduced mPAP and PVR in a patient subset	Not separately reported	Metabolic rather than redox target engagement	Small uncontrolled human study; response associated with SIRT3/UCP2 genotype
eNOS/BH4/DHFR recoupling	Mouse, rat and newborn pig models [26,27,134,135,136]	Both prevention and reversal (DHFR delivery after induction)	Prevented or reversed experimental PH	Not separately reported	Yes; improved eNOS coupling with endothelial-specific genetic evidence	None
eNOS–ER–mitochondrial axis (folic acid, PBA, MitoTEMPO)	DAHP- and hypoxia-induced mouse PH [25]	Concurrent with disease induction	Reduced pulmonary pressures and vascular remodeling	Not separately reported	Yes; reduced total and mitochondrial superoxide while preserving NO bioavailability	None
BH4 supplementation (sapropterin)	18 patients with PAH or inoperable CTEPH; open-label dose escalation [137]	Established disease	Not assessed	Not assessed	Not demonstrated; no significant change in NO synthesis or oxidant stress markers	Early human; phase I, small, uncontrolled, phenotypically mixed
Nitrate–nitrite–NO pathway	Mouse chronic hypoxia and bleomycin PH [69]	Both prevention and reversal of established hypoxic PH	Prevented hypoxic and bleomycin-associated PH; reversed established hypoxic PH	Not separately reported	Partial; effects attenuated by eNOS deletion or XOR inhibition	None
Inhaled sGC activator (mosliciguat)	38 participants with PAH or CTEPH; open-label phase 1b ATMOS trial [131]	Single dose in established disease	Mean peak PVR reduction of 21–38% across 0.24–4.0 mg, sustained over 3 h	Not assessed	Acts on the oxidized, heme-free form of sGC in smooth muscle, downstream of the endothelium	Early human; phase 1b, open-label, single-dose, uncontrolled
Broad antioxidants (TEMPOL, NAC)	Rat chronic hypoxia or SU5416/hypoxia [104,138]	TEMPOL concurrent; NAC compared early vs late	TEMPOL lowered RVSP without improving remodeling and worsened medial and adventitial remodeling in the severe model; NAC effective early and weaker late	Not separately reported	None; non-targeted scavengers	None
NRF2 activation (bardoxolone methyl)	Phase 2 LARIAT PAH cohort; phase 3 CATALYST CTD-PAH [139,140]	Established disease	LARIAT showed an early 6MWD signal; CATALYST ended before confirmatory efficacy	Not assessed	Not demonstrated in pulmonary endothelium	Human; preliminary phase 2 signal without phase 3 confirmation
Intravenous iron	Two randomized crossover trials in idiopathic or heritable PAH [108]	Established disease	No improvement in cardiopulmonary hemodynamics at 12 weeks	Not assessed	None; corrects systemic iron status, not the intracellular labile pool	Randomized human; no demonstrated clinical benefit
Ferroptosis inhibition (ferrostatin-1)	Rat monocrotaline and mouse SU5416/hypoxia PH [36,38,39]	Concurrent with disease induction	Reduced endothelial injury and pulmonary vascular remodeling across models	Not separately reported	Yes; restored GPX4 and FTH1 and reduced the labile iron pool in PAECs	None; human genetic and transcriptomic association only

Abbreviations: 6MWD, 6-minute walk distance; ATF4, activating transcription factor 4; BH4, tetrahydrobiopterin; CTD-PAH, connective tissue disease-associated pulmonary arterial hypertension; CTEPH, chronic thromboembolic pulmonary hypertension; DAHP, 2,4-diamino-6-hydroxypyrimidine; DCA, dichloroacetate; DHFR, dihydrofolate reductase; eIF2α, eukaryotic initiation factor 2 alpha; eNOS, endothelial nitric oxide synthase; ER, endoplasmic reticulum; FTH1, ferritin heavy chain 1; GPX4, glutathione peroxidase 4; HPAEC, human pulmonary artery endothelial cell; HPASMC, human pulmonary artery smooth muscle cell; HPV, hypoxic pulmonary vasoconstriction; IPAH, idiopathic pulmonary arterial hypertension; mPAP, mean pulmonary artery pressure; NAC, N-acetylcysteine; NO, nitric oxide; NOX, NADPH oxidase; NRF2, nuclear factor erythroid 2-related factor 2; PA, pulmonary artery; PAEC, pulmonary arterial endothelial cell; PAH, pulmonary arterial hypertension; PBA, phenylbutyric acid; PDK, pyruvate dehydrogenase kinase; PERK, protein kinase R-like endoplasmic reticulum kinase; PH, pulmonary hypertension; PVR, pulmonary vascular resistance; RV, right ventricle; RVSP, right ventricular systolic pressure; sGC, soluble guanylate cyclase; SIRT3, sirtuin 3; SOD2, superoxide dismutase 2; UCP2, uncoupling protein 2; XOR, xanthine oxidoreductase.

## 10. Conclusions and Research Priorities

Endothelial redox dysregulation in PAH is best understood as a failure of spatial and temporal control rather than a uniform increase in ROS. The strongest evidence implicates NOX-dependent signaling, mitochondrial dysfunction, and eNOS uncoupling, each supported in patient-derived cells or human tissue and in experimental models (Table 1), with growing but still experimental support for iron-dependent chemistry. Nuclear redox pathways, additional organelle sources, and EV-mediated communication remain considerably less established in human pulmonary endothelium. This framework helps explain why the same reactive species can support normal endothelial signaling in one context yet contribute to dysfunction in another. The biological outcome depends on the signal’s origin, which molecular targets are exposed, and how mechanical, metabolic, and inflammatory conditions shape the response.

Because these compartments are coupled through oxidase–mitochondrial amplification, H_2_O_2_ transport and buffering, and the eNOS–ER–mitochondrial circuit, interventions directed at a single source may be buffered by the others. Determining which nodes in this network are sufficient targets and which require combined intervention is a tractable experimental question and, in our view, a more productive one than further characterization of individual sources in isolation.

The major remaining gap is translation of these mechanisms to human PAH endothelium. Many redox pathways are supported by strong experimental data but have not been linked directly to a defined oxidant source, molecular target, and endothelial phenotype in patient-derived cells or diseased pulmonary vessels. Table 1 makes this evidence distribution explicit and highlights where direct disease-specific evidence remains limited.

Redox phenotyping, in the sense of matching a patient to an intervention directed at their predominant abnormality, is not currently achievable with available circulating markers, none of which is anchored to a specific oxidant source or compartment (Table 2). It would require measurements made in the relevant tissue or cells, whether patient-derived PAECs, explanted vessels, or a circulating marker whose relationship to a defined endothelial redox pathway has been demonstrated rather than assumed. Developing such measurements is a prerequisite for compartment-directed therapy, not a parallel activity.

Future work should also recognize that redox dysfunction is unlikely to be uniform across patients or disease stages. Studies should prioritize human validation, demonstrate target engagement at the proposed redox step, and determine whether correcting the selected redox defect improves pulmonary vascular function without disrupting adaptive signaling. The next phase of the field should move beyond asking whether oxidative stress is increased in PAH and instead define which redox pathway is abnormal, where it operates, and in which patients it presents a tractable therapeutic target.

## Figures and Tables

**Figure 3:**
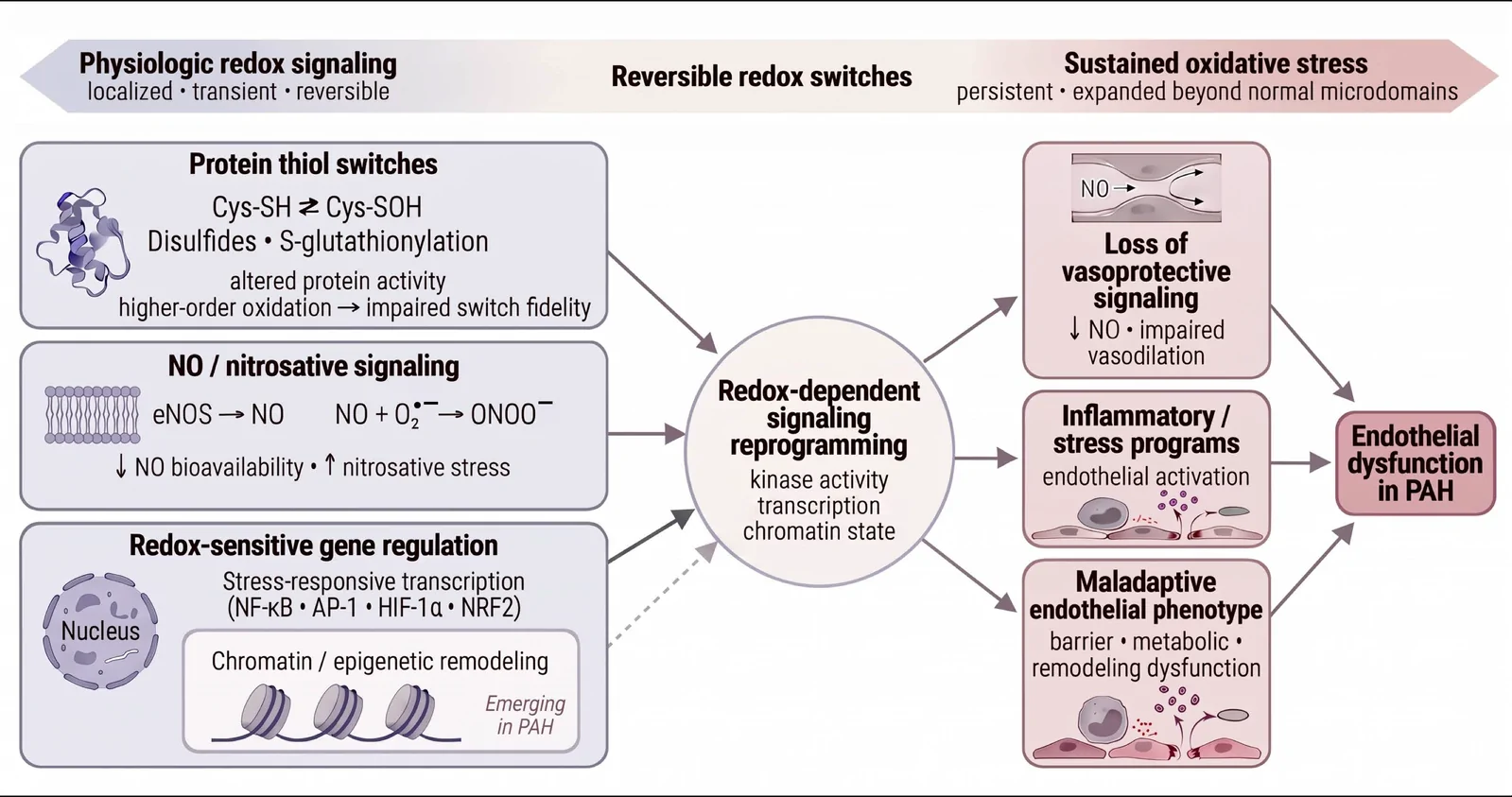
Redox switches couple oxidative stress to endothelial signaling reprogramming in PAH. Physiologic redox signaling is localized, transient, and reversible, whereas sustained oxidative stress promotes persistent and poorly compartmentalized oxidant signaling. Redox-sensitive mechanisms include reversible protein thiol modifications, altered nitric oxide bioavailability and nitrosative signaling, and changes in stress-responsive transcription. Chromatin and epigenetic remodeling are shown as emerging PAH mechanisms because their direct role in human PAH endothelium remains incompletely established. As oxidative stress becomes sustained, higher-order cysteine oxidation, reduced nitric oxide bioavailability, and dysregulated redox signaling can impair signaling fidelity and promote inflammatory, metabolic, barrier-disruptive, and pro-remodeling endothelial programs. Together, these changes drive redox-dependent signaling reprogramming and contribute to endothelial dysfunction in PAH. The dashed pathway denotes the emerging chromatin/epigenetic branch. Upward (↑) and downward (↓) arrows indicate increases and decreases, respectively. Created in BioRender. Marciano, D. (2026). https://BioRender.com/0vfwdws.

**Table 1:** Evidence hierarchy for endothelial redox pathways discussed in this review.

Pathway	Human PAH Vascular Tissue	Patient-Derived PAECs	Experimental PH	Pulmonary Endothelial Models	Extrapolated from Other Systems
Endothelial NOX1 signaling	[11]	[12]	[11]	[11]	-
NOX4 (pathogenic role)	[13]	-	[13,14]	-	-
NOX4 (adaptive role)	-	-	-	[15]	[16]
NOX5	-	-	-	-	[17]
Mitochondrial ROS production	-	[18]	[18,19,20]	[18,19]	[21,22]
SOD2 epigenetic attenuation (PASMC precedent)	[23]	-	[23]	-	-
eNOS uncoupling	[24]	-	[25,26,27]	[28]	[29,30,31]
Peroxynitrite and protein nitration	[32]	-	[33,34]	[35]	-
Labile iron and Fenton chemistry	-	-	[36] (labile iron)	-	[37] (Fenton chemistry)
Ferroptosis	-	-	[36,38,39]	[38]	[40,41]
Nuclear NOX4	-	-	-	-	[42]
8-oxoG and OGG1 signaling	-	-	-	-	[43,44,45,46]
TET and JmjC redox dependence	-	-	-	-	[47,48,49]
Endothelial enhancer remodeling	-	[50]	-	-	[51,52]
Shear-dependent redox signaling	-	[53,54]	[55,56]	[15,57,58]	[59,60,61,62,63]
Aquaporin-facilitated H_2_O_2_ transport	-	-	-	-	[64,65]
NOX-mitochondrial crosstalk	-	-	[19,36]	-	[66,67]
ROS-induced ROS release	-	-	-	-	[68]
Xanthine oxidoreductase	-	-	[69]	-	[70]
Peroxisomes, CYP, ER folding, lipoxygenases	-	-	-	-	[71,72,73,74,75]

Note. Each pathway is classified across five evidence levels, including human PAH vascular tissue, patient-derived PAECs, experimental pulmonary hypertension, pulmonary endothelial models without a PAH genotype or exposure, and extrapolation from non-pulmonary or non-endothelial systems. Numbers are reference citations, and a dash indicates that no evidence at that level was identified. Clinical, circulating, computational, and genetic evidence that cannot be assigned to vascular tissue is discussed in the text and is not counted as human vascular tissue evidence in this table. Abbreviations: CYP, cytochrome P450; eNOS, endothelial nitric oxide synthase; ER, endoplasmic reticulum; H_2_O_2_, hydrogen peroxide; JmjC, Jumonji C domain; NOX, NADPH oxidase; OGG1, 8-oxoguanine DNA glycosylase 1; 8-oxoG, 8-oxoguanine; PAECs, pulmonary arterial endothelial cells; PAH, pulmonary arterial hypertension; PASMC, pulmonary artery smooth muscle cell; PH, pulmonary hypertension; ROS, reactive oxygen species; SOD2, superoxide dismutase 2; TET, ten-eleven translocation enzymes.

## Data Availability

No new data were created or analyzed in this study. Data sharing is not applicable to this article.

## Abbreviations

8-oxoG, 8-oxoguanine; 8-OHdG, 8-hydroxy-2′-deoxyguanosine; 6MWD, 6-minute walk distance; ACE2, angiotensin-converting enzyme 2; ADMA, asymmetric dimethylarginine; AP-1, activator protein 1; APE1/Ref-1, apurinic/apyrimidinic endonuclease 1/redox factor 1; AQP3, aquaporin 3; ATF4, activating transcription factor 4; BH4, tetrahydrobiopterin; BMPR2, bone morphogenetic protein receptor type 2; cGMP, cyclic guanosine monophosphate; CHD-PAH, congenital heart disease-associated pulmonary arterial hypertension; CTD-PAH, connective tissue disease-associated pulmonary arterial hypertension; CTEPH, chronic thromboembolic pulmonary hypertension; CYP, cytochrome P450; DAHP, 2,4-diamino-6-hydroxypyrimidine; DCA, dichloroacetate; DHFR, dihydrofolate reductase; Drp1, dynamin-related protein 1; eIF2α, eukaryotic initiation factor 2 alpha; eNOS, endothelial nitric oxide synthase; ER, endoplasmic reticulum; ERG, ETS-related gene transcription factor; EV, extracellular vesicle; FTH1, ferritin heavy chain 1; GPX4, glutathione peroxidase 4; GPx8, glutathione peroxidase 8; H_2_O_2_, hydrogen peroxide; HIF, hypoxia-inducible factor; HPAEC, human pulmonary artery endothelial cell; HPASMC, human pulmonary artery smooth muscle cell; HPMVEC, human pulmonary microvascular endothelial cell; HPV, hypoxic pulmonary vasoconstriction; IPAH, idiopathic pulmonary arterial hypertension; JmjC, Jumonji C domain; KEAP1, Kelch-like ECH-associated protein 1; KLF2/KLF4, Krüppel-like factor 2/4; mPAP, mean pulmonary artery pressure; MISEV2023, Minimal Information for Studies of Extracellular Vesicles 2023; NAC, N-acetylcysteine; NADPH, nicotinamide adenine dinucleotide phosphate; NF-κB, nuclear factor kappa B; NO, nitric oxide; NOS3, nitric oxide synthase 3 gene; NOX, NADPH oxidase; NRF2, nuclear factor erythroid 2-related factor 2; NT-proBNP, N-terminal pro-brain natriuretic peptide; OGG1, 8-oxoguanine DNA glycosylase 1; PAEC, pulmonary arterial endothelial cell; PAH, pulmonary arterial hypertension; PASMC, pulmonary artery smooth muscle cell; PBA, phenylbutyric acid; PECAM-1, platelet endothelial cell adhesion molecule 1; PERK, protein kinase R-like endoplasmic reticulum kinase; PDK, pyruvate dehydrogenase kinase; PH, pulmonary hypertension; PPAR-γ, peroxisome proliferator-activated receptor gamma; PVR, pulmonary vascular resistance; ROS, reactive oxygen species; RV, right ventricle; RVSP, right ventricular systolic pressure; sGC, soluble guanylate cyclase; SHP2, Src homology 2 domain-containing phosphatase 2; SIRT3, sirtuin 3; SOD2, superoxide dismutase 2; SWI/SNF, switch/sucrose non-fermentable chromatin remodeling complex; TET, ten-eleven translocation enzymes; TGF-β, transforming growth factor beta; TNF, tumor necrosis factor; TNIK, TRAF2 and NCK-interacting kinase; TRPV4, transient receptor potential vanilloid 4; UCP2, uncoupling protein 2; UPR, unfolded protein response; VE-cadherin, vascular endothelial cadherin; XOR, xanthine oxidoreductase.
